# Poly[bis­(1,3-dimethyl-1,3-diazinan-2-one)(2,5-dioxidoterephthalato)zirconium(IV)]

**DOI:** 10.1107/S1600536813003437

**Published:** 2013-02-16

**Authors:** Matthias Maercz, David Stephen Wragg, Pascal Daniel Croumbie Dietzel, Helmer Fjellvåg

**Affiliations:** a Centre for Materials Science and Nanotechnology, Department of Chemistry, University of Oslo, PO Box 1126, 0315 Oslo, Norway; b Centre for Materials Science and Nanotechnology &, inGAP National Centre of Research-based Innovation, Department of Chemistry, University of Oslo, PO Box 1126, 0315 Oslo, Norway; c Department of Chemistry, University of Bergen, PO Box 7803, 5020 Bergen, Norway

## Abstract

In the title coordination polymer, [Zr(C_8_H_2_O_6_)(C_6_H_12_N_2_O)_2_]_*n*_, the Zr^IV^ atom, which lies on a crystallographic twofold rotation axis, is coordinated by two *O*,*O*′-bidentate 2,5-dioxidoterephthalate (DHTP^4−^) ligands and two *O*-bonded 1,3-dimethyl-1,3-diazinan-2-one (DMPU) ligands (the latter in a *cis* orientation) in a distorted ZrO_6_ octa­hedral geometry. The deprotonated hy­droxy and carb­oxy O atoms of the DHTP^4−^ ligand chelate the Zr^IV^ ion *via* a six-membered ring; the dihedral angle between the carboxyl­ate group and the aromatic ring is 19.94 (11)°. The DHTP^4−^ ligand is completed by crystallographic inversion symmetry and coordinates to two Zr^IV^ atoms, thereby forming polymeric zigzag chains propagating in the *c*-axis direction.

## Related literature

For examples of DHTP-containing MOFs, see: Dietzel *et al.* (2005 ▶, 2006 ▶). For examples of zirconium MOFs, see: Chavan *et al.* (2012 ▶). For a related structure, see: Maercz *et al.* (2013 ▶).
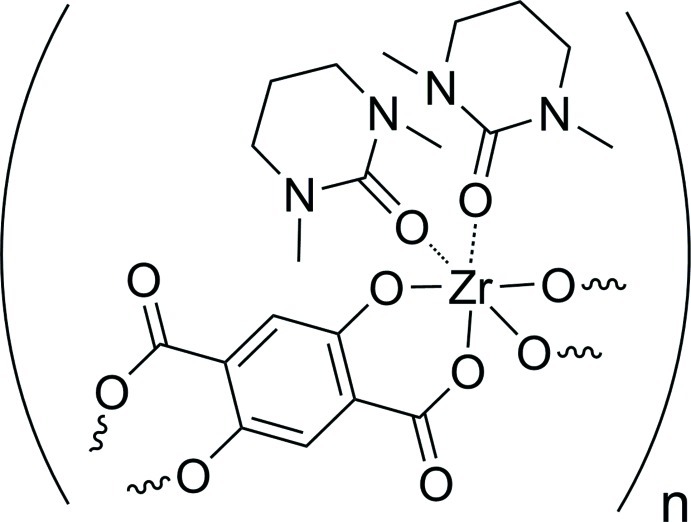


## Experimental

### 

#### Crystal data

[Zr(C_8_H_2_O_6_)(C_6_H_12_N_2_O)_2_]
*M* *_r_* = 541.67
Monoclinic, 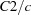

*a* = 18.761 (2) Å
*b* = 8.4049 (10) Å
*c* = 14.8816 (17) Å
β = 102.239 (1)°
*V* = 2293.3 (5) Å^3^
*Z* = 4
Mo *K*α radiation
μ = 0.53 mm^−1^
*T* = 293 K
0.10 × 0.08 × 0.06 mm

#### Data collection

Bruker SMART CCD diffractometer
Absorption correction: multi-scan (*SADABS*; Bruker, 2003 ▶) *T*_min_ = 0.949, *T*_max_ = 0.969
8477 measured reflections
2730 independent reflections
2437 reflections with *I* > 2σ(*I*)
*R*_int_ = 0.024

#### Refinement

*R*[*F* ^2^ > 2σ(*F* ^2^)] = 0.038
*wR*(*F* ^2^) = 0.096
*S* = 1.07
2730 reflections
150 parameters
H-atom parameters constrained
Δρ_max_ = 0.39 e Å^−3^
Δρ_min_ = −0.32 e Å^−3^

### 

Data collection: *SMART* (Bruker, 2003 ▶); cell refinement: *SAINT* (Bruker, 2003 ▶); data reduction: *SAINT*; program(s) used to solve structure: *SHELXS97* (Sheldrick, 2008 ▶); program(s) used to refine structure: *SHELXL97* (Sheldrick, 2008 ▶); molecular graphics: *DIAMOND* (Brandenburg, 2006 ▶); software used to prepare material for publication: *publCIF* (Westrip, 2010 ▶).

## Supplementary Material

Crystal structure: contains datablock(s) I, global. DOI: 10.1107/S1600536813003437/hb7026sup1.cif

Structure factors: contains datablock(s) I. DOI: 10.1107/S1600536813003437/hb7026Isup2.hkl

Additional supplementary materials:  crystallographic information; 3D view; checkCIF report

## Figures and Tables

**Table 1:** Selected bond lengths (Å)

Zr1—O1	2.0955 (18)
Zr2—O2	2.0723 (18)
Zr3—O3	2.0203 (17)

**Fig. 1.**
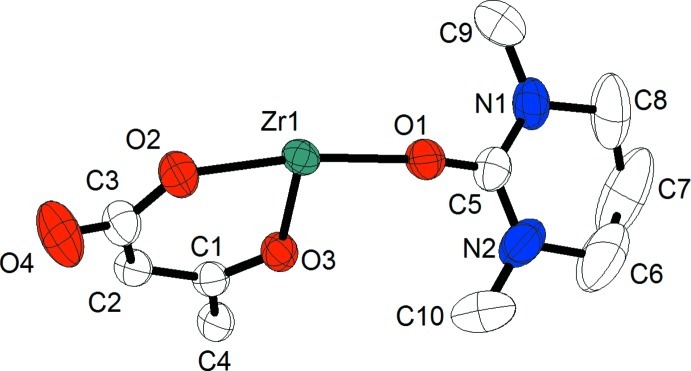
The asymmetric unit of the title compound with 50% probability displacement ellipsoids. Hydrogen atoms are omitted for clarity.

**Fig. 2.**
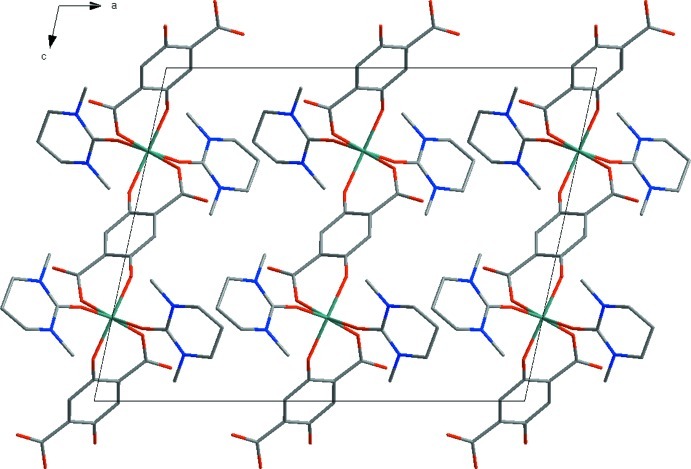
Packing diagram of the title compound viewed along the b axis. Hydrogen atoms are omitted for clarity.

## supplementary crystallographic information

### Comment

The title compound was synthesized as a part of a larger project (see also Maercz *et al.*, 2013), in which the possibility to form new zirconium containing metal organic frameworks (MOFs) using the linker 2,5-dihydroxyterephthalic acid (DHTP) was investigated. Zirconium containing MOFs (Chavan *et al.*, 2012) using terephthalic acid have shown extraordinary thermal stability whereas DHTP containing MOFs (Dietzel *et al.*, 2005, 2006) have shown remarkable sorption properties.

### Experimental

Zirconium(IV) chloride (0.047 g, 0.2 mmol) and 2,5-dihydroxyterephthalic acid (0.040 g, 0.2 mmol) were dissolved in 5 ml *N,N'*-dimethylpropylene urea (DMPU) in a Teflon liner of 23 ml volume under inert atmosphere in a glovebox. The teflon liner was sealed and put into a steel autoclave. The mixture was reacted for 3 d at 160°C. Reaction yielded a brown substance consisting of block shaped crystals and a white impurity. The product was collected by filtration, washed with DMPU and dried over night at room temperature in ambient atmosphere.

### Refinement

Hydrogen atoms were placed geometrically in ideal positions and refined using a riding model, the *U*_iso_ set to 1.5 times the thermal parameter of the carbon atom to which they are attached for methyl groups and 1.2 times for other hydrogen atoms.

### Crystal data

**Table:**

[Zr(C_8_H_2_O_6_)(C_6_H_12_N_2_O)_2_]	*F*(000) = 1112
*M**_r_* = 541.67	*D*_x_ = 1.569 Mg m^−^^3^
Monoclinic, *C*2/*c*	Mo *K*α radiation, λ = 0.71073 Å
*a* = 18.761 (2) Å	Cell parameters from 2840 reflections
*b* = 8.4049 (10) Å	θ = 2.8–24.3°
*c* = 14.8816 (17) Å	µ = 0.53 mm^−^^1^
β = 102.239 (1)°	*T* = 293 K
*V* = 2293.3 (5) Å^3^	Block, colourless
*Z* = 4	0.10 × 0.08 × 0.06 mm

### Data collection

**Table:**

Bruker SMART CCD diffractometer	2730 independent reflections
Radiation source: fine-focus sealed tube	2437 reflections with *I* > 2σ(*I*)
Graphite monochromator	*R*_int_ = 0.024
phi and ω scans	θ_max_ = 28.8°, θ_min_ = 2.2°
Absorption correction: multi-scan (*SADABS*; Bruker, 2003)	*h* = −24→25
*T*_min_ = 0.949, *T*_max_ = 0.969	*k* = −10→10
8477 measured reflections	*l* = −19→20

### Refinement

**Table:**

Refinement on *F*^2^	Primary atom site location: structure-invariant direct methods
Least-squares matrix: full	Secondary atom site location: difference Fourier map
*R*[*F*^2^ > 2σ(*F*^2^)] = 0.038	Hydrogen site location: inferred from neighbouring sites
*wR*(*F*^2^) = 0.096	H-atom parameters constrained
*S* = 1.07	*w* = 1/[σ^2^(*F*_o_^2^) + (0.0462*P*)^2^ + 2.0694*P*] where *P* = (*F*_o_^2^ + 2*F*_c_^2^)/3
2730 reflections	(Δ/σ)_max_ < 0.001
150 parameters	Δρ_max_ = 0.39 e Å^−^^3^
0 restraints	Δρ_min_ = −0.32 e Å^−^^3^

### Special details

**Table:**

Geometry. All s.u.'s (except the s.u. in the dihedral angle between two l.s. planes) are estimated using the full covariance matrix. The cell s.u.'s are taken into account individually in the estimation of s.u.'s in distances, angles and torsion angles; correlations between s.u.'s in cell parameters are only used when they are defined by crystal symmetry. An approximate (isotropic) treatment of cell s.u.'s is used for estimating s.u.'s involving l.s. planes.
Refinement. Refinement of *F*^2^ against ALL reflections. The weighted *R*-factor *wR* and goodness of fit *S* are based on *F*^2^, conventional *R*-factors *R* are based on *F*, with *F* set to zero for negative *F*^2^. The threshold expression of *F*^2^ > 2σ(*F*^2^) is used only for calculating *R*-factors(gt) *etc*. and is not relevant to the choice of reflections for refinement. *R*-factors based on *F*^2^ are statistically about twice as large as those based on *F*, and *R*- factors based on ALL data will be even larger.

### Fractional atomic coordinates and isotropic or equivalent isotropic displacement parameters (Å^2^)

**Table:**

	*x*	*y*	*z*	*U*_iso_*/*U*_eq_	
C1	0.01168 (12)	0.8750 (3)	0.06523 (15)	0.0370 (5)	
C2	−0.04708 (11)	0.9809 (3)	0.06010 (16)	0.0389 (5)	
C3	−0.10053 (13)	0.9651 (4)	0.12208 (19)	0.0494 (6)	
C4	0.05755 (12)	0.8979 (3)	0.00446 (16)	0.0409 (5)	
H4	0.0968	0.8292	0.0072	0.049*	
C5	0.13245 (14)	0.4514 (3)	0.28509 (18)	0.0460 (6)	
C6	0.1951 (3)	0.2463 (6)	0.2172 (4)	0.1152 (19)	
H6A	0.1811	0.1438	0.2380	0.138*	
H6B	0.2043	0.2328	0.1559	0.138*	
C7	0.2608 (3)	0.3007 (6)	0.2785 (6)	0.137 (3)	
H7A	0.2813	0.3874	0.2491	0.164*	
H7B	0.2961	0.2146	0.2880	0.164*	
C8	0.25001 (19)	0.3551 (5)	0.3685 (4)	0.0998 (15)	
H8A	0.2447	0.2635	0.4061	0.120*	
H8B	0.2927	0.4139	0.3993	0.120*	
C9	0.1770 (2)	0.5490 (5)	0.4385 (2)	0.0860 (12)	
H9A	0.2201	0.5396	0.4862	0.129*	
H9B	0.1357	0.5104	0.4607	0.129*	
H9C	0.1694	0.6587	0.4211	0.129*	
C10	0.0730 (3)	0.3413 (7)	0.1388 (3)	0.1198 (18)	
H10A	0.0849	0.2712	0.0932	0.180*	
H10B	0.0594	0.4435	0.1117	0.180*	
H10C	0.0331	0.2977	0.1617	0.180*	
N1	0.18563 (12)	0.4566 (3)	0.35990 (19)	0.0583 (6)	
N2	0.13613 (17)	0.3589 (3)	0.2142 (2)	0.0732 (8)	
O1	0.07673 (10)	0.5374 (2)	0.28218 (12)	0.0532 (5)	
O2	−0.07967 (9)	0.8873 (2)	0.19808 (13)	0.0519 (5)	
O3	0.02342 (9)	0.7526 (2)	0.12481 (12)	0.0424 (4)	
O4	−0.16069 (11)	1.0250 (4)	0.10048 (17)	0.0870 (9)	
Zr1	0.0000	0.72101 (4)	0.2500	0.03776 (12)	

### Atomic displacement parameters (Å^2^)

**Table:**

	*U*^11^	*U*^22^	*U*^33^	*U*^12^	*U*^13^	*U*^23^
C1	0.0321 (10)	0.0424 (14)	0.0359 (11)	0.0017 (9)	0.0060 (8)	−0.0032 (9)
C2	0.0295 (10)	0.0489 (15)	0.0387 (12)	0.0026 (10)	0.0086 (9)	−0.0011 (10)
C3	0.0397 (13)	0.0587 (17)	0.0531 (15)	0.0122 (12)	0.0173 (11)	0.0085 (12)
C4	0.0306 (10)	0.0495 (15)	0.0432 (12)	0.0076 (10)	0.0095 (9)	0.0001 (10)
C5	0.0470 (13)	0.0399 (14)	0.0542 (15)	0.0022 (11)	0.0176 (11)	0.0035 (11)
C6	0.145 (5)	0.082 (3)	0.140 (5)	0.039 (3)	0.078 (4)	−0.006 (3)
C7	0.083 (3)	0.077 (3)	0.277 (9)	0.013 (2)	0.098 (5)	−0.021 (4)
C8	0.0489 (19)	0.069 (3)	0.169 (5)	0.0175 (18)	−0.004 (2)	0.017 (3)
C9	0.086 (2)	0.094 (3)	0.062 (2)	0.013 (2)	−0.0200 (18)	−0.0037 (19)
C10	0.172 (5)	0.107 (4)	0.069 (3)	0.012 (4)	0.001 (3)	−0.037 (3)
N1	0.0422 (12)	0.0490 (15)	0.0787 (17)	0.0082 (10)	0.0014 (11)	0.0044 (12)
N2	0.097 (2)	0.0551 (17)	0.0735 (18)	0.0136 (16)	0.0320 (16)	−0.0102 (14)
O1	0.0458 (10)	0.0629 (13)	0.0478 (10)	0.0151 (9)	0.0028 (8)	−0.0043 (9)
O2	0.0445 (9)	0.0666 (13)	0.0492 (10)	0.0150 (9)	0.0205 (8)	0.0134 (9)
O3	0.0408 (9)	0.0467 (11)	0.0410 (9)	0.0073 (7)	0.0117 (7)	0.0035 (7)
O4	0.0529 (12)	0.134 (2)	0.0840 (16)	0.0459 (14)	0.0373 (11)	0.0487 (16)
Zr1	0.03075 (17)	0.0470 (2)	0.03504 (18)	0.000	0.00586 (12)	0.000

### Geometric parameters (Å, º)

**Table:**

C1—O3	1.346 (3)	C7—H7B	0.9700
C1—C4	1.388 (3)	C8—N1	1.462 (4)
C1—C2	1.406 (3)	C8—H8A	0.9700
C2—C4^i^	1.386 (3)	C8—H8B	0.9700
C2—C3	1.505 (3)	C9—N1	1.442 (4)
C3—O4	1.215 (3)	C9—H9A	0.9600
C3—O2	1.293 (3)	C9—H9B	0.9600
C4—C2^i^	1.386 (3)	C9—H9C	0.9600
C4—H4	0.9300	C10—N2	1.456 (5)
C5—O1	1.264 (3)	C10—H10A	0.9600
C5—N2	1.325 (4)	C10—H10B	0.9600
C5—N1	1.329 (3)	C10—H10C	0.9600
C6—C7	1.443 (8)	Zr1—O1	2.0955 (18)
C6—N2	1.449 (5)	Zr2—O2	2.0723 (18)
C6—H6A	0.9700	Zr3—O3	2.0203 (17)
C6—H6B	0.9700	Zr1—O3^ii^	2.0203 (17)
C7—C8	1.470 (8)	Zr1—O2^ii^	2.0723 (18)
C7—H7A	0.9700	Zr1—O1^ii^	2.0955 (18)
			
O3—C1—C4	119.9 (2)	H9A—C9—H9B	109.5
O3—C1—C2	122.5 (2)	N1—C9—H9C	109.5
C4—C1—C2	117.7 (2)	H9A—C9—H9C	109.5
C4^i^—C2—C1	119.8 (2)	H9B—C9—H9C	109.5
C4^i^—C2—C3	118.3 (2)	N2—C10—H10A	109.5
C1—C2—C3	121.8 (2)	N2—C10—H10B	109.5
O4—C3—O2	122.2 (2)	H10A—C10—H10B	109.5
O4—C3—C2	120.2 (2)	N2—C10—H10C	109.5
O2—C3—C2	117.6 (2)	H10A—C10—H10C	109.5
C2^i^—C4—C1	122.5 (2)	H10B—C10—H10C	109.5
C2^i^—C4—H4	118.7	C5—N1—C9	120.3 (2)
C1—C4—H4	118.7	C5—N1—C8	120.9 (3)
O1—C5—N2	119.4 (3)	C9—N1—C8	118.4 (3)
O1—C5—N1	118.6 (2)	C5—N2—C6	121.8 (4)
N2—C5—N1	122.0 (3)	C5—N2—C10	120.2 (3)
C7—C6—N2	110.9 (4)	C6—N2—C10	116.7 (4)
C7—C6—H6A	109.5	C5—O1—Zr1	162.17 (19)
N2—C6—H6A	109.5	C3—O2—Zr1	136.71 (16)
C7—C6—H6B	109.5	C1—O3—Zr1	132.13 (15)
N2—C6—H6B	109.5	O3—Zr1—O3^ii^	164.90 (10)
H6A—C6—H6B	108.0	O3—Zr1—O2^ii^	88.54 (7)
C6—C7—C8	114.2 (4)	O3^ii^—Zr1—O2^ii^	81.27 (7)
C6—C7—H7A	108.7	O3—Zr1—O2	81.27 (7)
C8—C7—H7A	108.7	O3^ii^—Zr1—O2	88.54 (7)
C6—C7—H7B	108.7	O2^ii^—Zr1—O2	95.19 (12)
C8—C7—H7B	108.7	O3—Zr1—O1^ii^	99.16 (7)
H7A—C7—H7B	107.6	O3^ii^—Zr1—O1^ii^	91.97 (7)
N1—C8—C7	111.9 (4)	O2^ii^—Zr1—O1^ii^	171.16 (7)
N1—C8—H8A	109.2	O2—Zr1—O1^ii^	90.30 (8)
C7—C8—H8A	109.2	O3—Zr1—O1	91.97 (7)
N1—C8—H8B	109.2	O3^ii^—Zr1—O1	99.16 (7)
C7—C8—H8B	109.2	O2^ii^—Zr1—O1	90.30 (8)
H8A—C8—H8B	107.9	O2—Zr1—O1	171.16 (7)
N1—C9—H9A	109.5	O1^ii^—Zr1—O1	85.13 (11)
N1—C9—H9B	109.5		
			
O3—C1—C2—C4^i^	−178.2 (2)	C7—C6—N2—C10	164.6 (5)
C4—C1—C2—C4^i^	0.3 (4)	N2—C5—O1—Zr1	−75.3 (7)
O3—C1—C2—C3	0.7 (4)	N1—C5—O1—Zr1	104.9 (6)
C4—C1—C2—C3	179.2 (2)	O4—C3—O2—Zr1	160.2 (3)
C4^i^—C2—C3—O4	20.1 (4)	C2—C3—O2—Zr1	−20.1 (4)
C1—C2—C3—O4	−158.8 (3)	C4—C1—O3—Zr1	152.68 (18)
C4^i^—C2—C3—O2	−159.5 (3)	C2—C1—O3—Zr1	−28.8 (3)
C1—C2—C3—O2	21.6 (4)	C1—O3—Zr1—O3^ii^	−23.03 (19)
O3—C1—C4—C2^i^	178.2 (2)	C1—O3—Zr1—O2^ii^	−70.4 (2)
C2—C1—C4—C2^i^	−0.3 (4)	C1—O3—Zr1—O2	25.1 (2)
N2—C6—C7—C8	47.7 (6)	C1—O3—Zr1—O1^ii^	114.0 (2)
C6—C7—C8—N1	−44.6 (6)	C1—O3—Zr1—O1	−160.7 (2)
O1—C5—N1—C9	4.8 (4)	C3—O2—Zr1—O3	0.6 (3)
N2—C5—N1—C9	−175.0 (3)	C3—O2—Zr1—O3^ii^	169.4 (3)
O1—C5—N1—C8	177.6 (3)	C3—O2—Zr1—O2^ii^	88.3 (3)
N2—C5—N1—C8	−2.2 (5)	C3—O2—Zr1—O1^ii^	−98.6 (3)
C7—C8—N1—C5	21.3 (5)	C3—O2—Zr1—O1	−39.9 (6)
C7—C8—N1—C9	−165.7 (4)	C5—O1—Zr1—O3	29.7 (6)
O1—C5—N2—C6	−173.7 (3)	C5—O1—Zr1—O3^ii^	−140.1 (6)
N1—C5—N2—C6	6.0 (5)	C5—O1—Zr1—O2^ii^	−58.9 (6)
O1—C5—N2—C10	−7.7 (5)	C5—O1—Zr1—O2	69.6 (9)
N1—C5—N2—C10	172.1 (4)	C5—O1—Zr1—O1^ii^	128.7 (6)
C7—C6—N2—C5	−28.8 (6)		

Symmetry codes: (i) −*x*, −*y*+2, −*z*; (ii) −*x*, *y*, −*z*+1/2.
